# Correction: Bähr et al. Repurposing Nitazoxanide for Potential Treatment of Rare Disease Lymphangioleiomyomatosis. *Biomolecules* 2024, *14*, 1236

**DOI:** 10.3390/biom16071005

**Published:** 2026-07-10

**Authors:** Stella Bähr, Ryan W. Rue, Carly J. Smith, Jillian F. Evans, Hubert Köster, Vera P. Krymskaya, Dirk Pleimes

**Affiliations:** 1 Faculty of Engineering Sciences, Heidelberg University, 69120 Heidelberg, Germany; 2 Biosputnik LLC., New York, NY 10002, USA; ryanrue@pennmedicine.upenn.edu (R.W.R.); carly.smith9620@gmail.com (C.J.S.); 3 Division of Pulmonary, Allergy, and Critical Care Medicine, Department of Medicine, University of Pennsylvania, Philadelphia, PA 19104, USA; jillyfevans@gmail.com (J.F.E.); krymskay@pennmedicine.upenn.edu (V.P.K.); 4 Emeritus Professor of Organic Chemistry and Biochemistry, University Hamburg, 20148 Hamburg, Germany; hubertkoster07@gmail.com

In the original publication [1], several errors were identified in Figure 5. To ensure accuracy, the panel was reassembled from the original source images. The following corrections were made relative to the original publication:

- NTZ Oral (500 mg/kg): The correct images for this condition were present in the original dataset but had been placed incorrectly. In the published figure, these images (both whole lung and magnified view) were mistakenly used for the condition NTZ i.p. (100 mg/kg) + Rapa i.p. (0.1 mg/kg). This has now been corrected.
- NTZ i.p. (100 mg/kg) + Rapa i.p. (0.1 mg/kg): As a result of the misassignment described above, the correct images for this condition were not properly represented in the original publication. The appropriate whole lung image was absent, and the magnified image had been incorrectly assigned to NTZ Oral (500 mg/kg). Both images have now been replaced with the correct ones.
- NTZ i.p. (100 mg/kg) and NTZ Oral (500 mg/kg) + Rapa i.p. (0.5 mg/kg): These two conditions were mislabeled in the original figure (titles interchanged). The corresponding whole lung and magnified images were otherwise correctly matched to their respective conditions. The labeling has now been corrected.

All figure panels have been verified following reconstruction to ensure that each image corresponds accurately to its labeled experimental condition. The authors state that the scientific conclusions are unaffected. This correction was approved by the Academic Editor. The original publication has also been updated.

**Figure 5:**
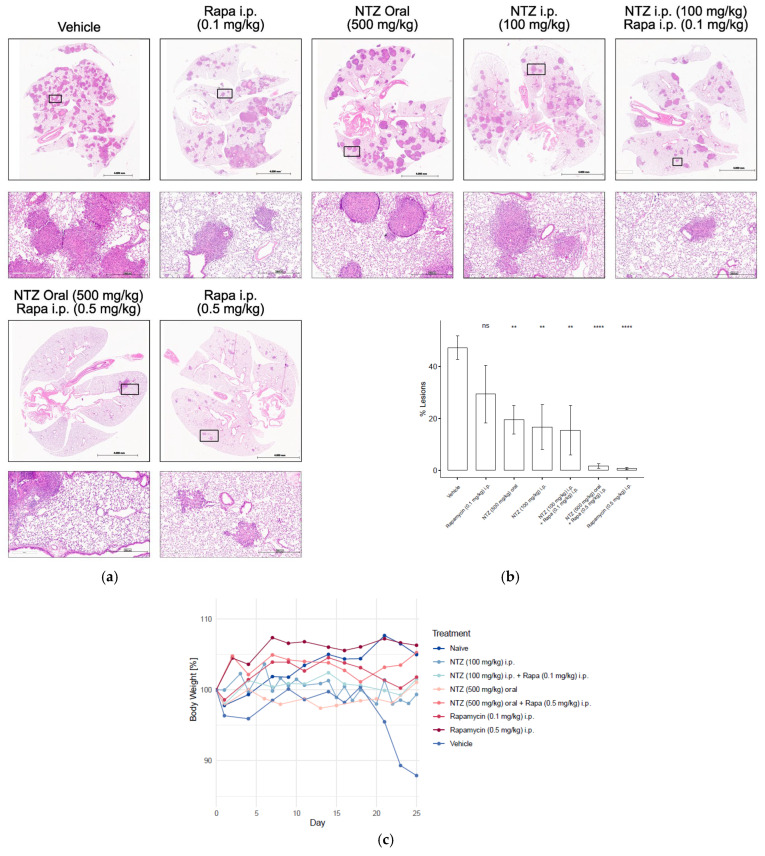
Representative H&E stainings, percent-lesions, and body weight changes in Tsc2-null lung lesions in a metastatic immunocompromised mouse model of LAM. Mice received 800,000 Tsc2-null TTJ cells injected into the tail vein. The animals received pharmacological treatment beginning 3–7 days after the injection of cells. Animals were euthanized 28 days after injection of the Tsc2-null cells. In two subsequent studies with a similar design, different NTZ and Rapamycin doses and combination were tested. (**a**) Lungs were inflated and stained with H&E. Scale bars: 4 mm and 500 μm. (**b**) Percent-lesion formation in lungs of the treated mice at takedown. Shown is an average of all mice in the treatment groups. (**c**) Development of body weight over the course of the study, presented as percentage change from baseline body weight on day 0. Group sizes were as follows: Vehicle n = 5, Rapa (0.1 mg/kg) i.p. n = 5, NTZ (500 mg/kg) oral n = 10, NTZ (100 mg/kg) i.p. + Rapa (0.1 mg/kg) i.p. n = 5, NTZ (100 mg/kg) i.p. n = 5, NTZ (500 mg/kg) oral + Rapa (0.5 mg/kg) i.p. n = 10, Rapa (0.5 mg/kg) i.p. n = 10. (ns non-significant, ** *p* < 0.01, **** *p* < 0.0001).
