# Supplementary material for: Repurposing Nitazoxanide for Potential Treatment of Rare Disease Lymphangioleiomyomatosis
Source: Biomolecules. 2024 Sep 30;14(10):1236. doi: 10.3390/biom14101236 (PMC11506457; doi:10.3390/biom14101236)
Supplement: Supplementary file 1 [file biomolecules-14-01236-s001.zip › biomolecules-3182956-supplementary.pdf]

Supplementary Table S1: IC 50 Raw Data Input, Response equals percent proliferation normalized by background.

Cell	Dose [µM]	Response
621_102	0	0.96165619
621_102	0	0.8572305
621_102	0	1.12482133
621_102	0	0.99428922
621_102	0	1.0856617
621_102	0	0.94860298
621_102	0	0.96165619
621_102	0	1.06608188
621_102	0	0.90385873
621_102	0	0.91955526
621_102	0	0.95618051
621_102	0	1.03989536
621_102	0	0.92478744
621_102	0	1.03466318
621_102	0	1.19686069
621_102	0	1.02419882
621_102	0	0.95570139
621_102	0	0.96882691
621_102	0	0.92288761
621_102	0	0.89663659
621_102	0	0.96882691
621_102	0	1.17227235
621_102	0	1.1197703
621_102	0	0.99507793

Cell	Dose [µM]	Response
621_102	5	0.82014388
621_102	5	0.77305428
621_102	5	0.79921517
621_102	5	0.72596468
621_102	5	0.84413454
621_102	5	0.71944217
621_102	5	0.75225595
621_102	5	0.75225595
621_102	10	0.793983
621_102	10	0.63178548
621_102	10	0.66841073
621_102	10	0.74166122
621_102	10	0.66694011
621_102	10	0.72600492
621_102	10	0.60787531
621_102	10	0.7588187
621_102	15	0.50621321
621_102	15	0.74166122
621_102	15	0.6736429
621_102	15	0.50621321
621_102	15	0.68662838
621_102	15	0.62100082
621_102	15	0.62100082
621_102	15	0.76538146

Cell	Dose [µM]	Response
621_102	30	-0.1796555
621_102	30	-0.3240361
621_102	30	-0.2518458
621_102	30	-0.3765381
621_102	30	-0.0693264
621_102	30	-0.0902551
621_102	30	-0.1635056
621_102	30	-0.116416
621_102	45	-0.3896637
621_102	45	-0.3765381
621_102	45	-0.2780968
621_102	45	-0.245283
621_102	45	-0.1268803
621_102	45	-0.2262917
621_102	45	-0.1687377
621_102	45	-0.1844343
621_102	50	-0.2846596
621_102	50	-0.3240361
621_102	50	-0.2518458
621_102	50	-0.3765381
621_102	50	-0.3045053
621_102	50	-0.2131328
621_102	50	-0.1413402
621_102	50	-0.1413402

Cell	Dose [µM]	Response
621_102	50	-0.1373447
621_102	50	-0.1582734
621_102	50	-0.1425768
621_102	50	-0.2576848
621_102	100	-0.5340443
621_102	100	-0.4684167
621_102	100	-0.3831009
621_102	100	-0.4027892
621_102	100	-0.0630209
621_102	100	-0.1674466
621_102	100	-0.226186
621_102	100	-0.2653457
621_102	100	-0.1007194
621_102	100	-0.1216481
621_102	100	-0.1111838
621_102	100	-0.2681491
621_103	0	1.13553497
621_103	0	1.17688463
621_103	0	0.94256993
621_103	0	0.97932517
621_103	0	1.0804021
621_103	0	0.83230419
621_103	0	0.93338112
621_103	0	0.9195979

Cell	Dose [µM]	Response
621_103	0	0.8992629
621_103	0	1.00933661
621_103	0	0.93857494
621_103	0	0.91891892
621_103	0	1.03292383
621_103	0	0.93464373
621_103	0	1.15479115
621_103	0	1.11154791
621_103	0	0.89240023
621_103	0	1.03227993
621_103	0	1.16139966
621_103	0	0.91392018
621_103	0	0.90316021
621_103	0	1.01075998
621_103	0	1.12911973
621_103	0	0.95696009
621_103	5	0.72235872
621_103	5	0.76167076
621_103	5	0.9031941
621_103	5	0.66339066
621_103	5	0.47276112
621_103	5	0.59112087
621_103	5	0.51580103
621_103	5	0.526561

Cell	Dose [µM]	Response
621_103	10	0.80884521
621_103	10	0.64766585
621_103	10	0.65159705
621_103	10	0.64373464
621_103	10	0.40820125
621_103	10	0.45124116
621_103	10	0.34364139
621_103	10	0.56960091
621_103	15	0.82457002
621_103	15	0.27813268
621_103	15	0.67125307
621_103	15	0.40786241
621_103	15	0.44048119
621_103	15	0.36516135
621_103	15	0.28984151
621_103	15	0.526561
621_103	30	-0.0167076
621_103	30	-0.004914
621_103	30	0.02653563
621_103	30	-0.044226
621_103	30	-0.6032366
621_103	30	-0.7108364
621_103	30	-0.5924766
621_103	30	-0.7108364

Cell	Dose [µM]	Response
621_103	45	0.05405405
621_103	45	-0.0481572
621_103	45	0.03046683
621_103	45	-0.0363636
621_103	45	-0.6785564
621_103	45	-0.6785564
621_103	45	-0.2804373
621_103	45	-0.6462765
621_103	50	-0.0498217
621_103	50	-0.1049546
621_103	50	-0.1233322
621_103	50	-0.0130665
621_103	50	-0.0402948
621_103	50	-0.044226
621_103	50	0.13660934
621_103	50	-0.0009828
621_103	50	-0.6677965
621_103	50	-0.6570365
621_103	50	-0.6893164
621_103	50	-0.7215964
621_103	100	-0.0992629
621_103	100	0.04619165
621_103	45	0.05405405
621_103	45	-0.0481572

Cell	Dose [µM]	Response
621_103	100	0.10515971
621_103	100	0.02653563
621_103	100	-0.0268497
621_103	100	-0.0727938
621_103	100	-0.1508987
621_103	100	-0.1003602
621_103	100	-0.5601967
621_103	100	-0.6677965
621_103	100	-0.6247566
621_103	100	-0.7000764
LAMD100	0	0.719
LAMD100	0	0.988
LAMD100	0	1.328
LAMD100	0	1.047
LAMD100	0	1.018
LAMD100	0	1.070
LAMD100	0	0.789
LAMD100	0	1.041
LAMD100	0	0.994
LAMD100	0	1.000
LAMD100	0	0.963
LAMD100	0	0.996
LAMD100	0	0.952
LAMD100	0	1.000

Cell	Dose [µM]	Response
LAMD100	0	0.96318516
LAMD100	0	0.99590946
LAMD100	0	0.95227707
LAMD100	0	1.0002727
LAMD100	0	1.12026179
LAMD100	0	0.97409326
LAMD100	0	0.98290995
LAMD100	0	1.00520132
LAMD100	0	1.04086752
LAMD100	0	0.94278547
LAMD100	0	1.00520132
LAMD100	0	0.98736822
LAMD100	0	1.00520132
LAMD100	0	1.03046488
LAMD100	5	1.18737447
LAMD100	5	0.59597379
LAMD100	5	0.56669653
LAMD100	5	0.46715384
LAMD100	5	0.70139078
LAMD100	5	0.74284156
LAMD100	5	0.68611944
LAMD100	5	0.70793564
LAMD100	5	0.93089674
LAMD100	5	0.91306364

Cell	Dose [µM]	Response
621_103	45	0.05405405
621_103	45	-0.0481572
621_103	45	0.03046683
621_103	45	-0.0363636
621_103	45	-0.6785564
621_103	45	-0.6785564
621_103	45	-0.2804373
621_103	45	-0.6462765
621_103	50	-0.0498217
621_103	50	-0.1049546
621_103	50	-0.1233322
621_103	50	-0.0130665
621_103	50	-0.0402948
621_103	50	-0.044226
621_103	50	0.13660934
621_103	50	-0.0009828
621_103	50	-0.6677965
621_103	50	-0.6570365
621_103	50	-0.6893164
621_103	50	-0.7215964
621_103	100	-0.0992629
621_103	100	0.04619165
621_103	45	0.05405405
621_103	45	-0.0481572

Cell	Dose [µM]	Response
LAMD100	5	0.94724375
LAMD100	5	0.902661
LAMD100	10	0.62525105
LAMD100	10	0.77749281
LAMD100	10	0.69551648
LAMD100	10	0.53156382
LAMD100	10	0.62503409
LAMD100	10	0.58140169
LAMD100	10	0.55522225
LAMD100	10	0.66648487
LAMD100	10	0.88185571
LAMD100	10	0.80903722
LAMD100	10	0.77039884
LAMD100	10	0.87293916
LAMD100	15	0.56669653
LAMD100	15	0.38517751
LAMD100	15	0.42031022
LAMD100	15	0.71308284
LAMD100	15	0.69484592
LAMD100	15	0.57049359
LAMD100	15	0.62067085
LAMD100	15	0.24325061
LAMD100	30	0.84775824
LAMD100	30	0.49057565

Cell	Dose [µM]	Response
LAMD100	30	0.71308284
LAMD100	30	0.93559002
LAMD100	30	0.68611944
LAMD100	30	0.70139078
LAMD100	30	0.33051541
LAMD100	30	-0.0447232
LAMD100	30	0.89225835
LAMD100	30	0.70055253
LAMD100	30	0.89968881
LAMD100	30	0.72135781
LAMD100	45	0.46715384
LAMD100	45	0.42031022
LAMD100	45	0.44958748
LAMD100	45	0.40859932
LAMD100	45	0.26070357
LAMD100	45	0.30215435
LAMD100	45	0.29124625
LAMD100	45	0.14071448
LAMD100	45	0.80012067
LAMD100	45	0.50736061
LAMD100	45	0.6708307
LAMD100	45	0.57720692
LAMD100	50	0.20365849
LAMD100	50	0.56084108

Cell	Dose [µM]	Response
LAMD100	50	0.51985291
LAMD100	50	0.63696196
LAMD100	50	0.38287428
LAMD100	50	0.17125716
LAMD100	50	0.1647123
LAMD100	50	0.19089174
LAMD100	100	0.14510396
LAMD100	100	0.21536939
LAMD100	100	0.32662298
LAMD100	100	0.15681487
LAMD100	100	-0.0447232
LAMD100	100	-0.0534497
LAMD100	100	-0.0949005
LAMD100	100	0.01199891
LAMHUP	0	0.71378968
LAMHUP	0	0.91220238
LAMHUP	0	1.23759921
LAMHUP	0	1.07490079
LAMHUP	0	1.13045635
LAMHUP	0	1.05505952
LAMHUP	0	0.9280754
LAMHUP	0	0.94791667
LAMHUP	0	0.86028225
LAMHUP	0	0.96479378

Cell	Dose [µM]	Response
LAMHUP	0	1.10650434
LAMHUP	0	1.08170499
LAMHUP	0	1.02147801
LAMHUP	0	0.94885135
LAMHUP	0	1.07993361
LAMHUP	0	0.93645167
LAMHUP	0	1.02825747
LAMHUP	0	0.99101391
LAMHUP	0	0.98062036
LAMHUP	0	1.00573811
LAMHUP	0	0.9936123
LAMHUP	0	1.00747037
LAMHUP	0	1.02392682
LAMHUP	0	0.96936068
LAMHUP	1	0.91825905
LAMHUP	1	0.79526868
LAMHUP	1	1.0351865
LAMHUP	1	0.77274932
LAMHUP	1	1.02679215
LAMHUP	1	0.85673949
LAMHUP	1	0.924052
LAMHUP	1	1.00376419
LAMHUP	5	0.64236111
LAMHUP	5	0.7296627

Cell	Dose [µM]	Response
LAMHUP	5	0.77331349
LAMHUP	5	0.51140873
LAMHUP	5	0.99490728
LAMHUP	5	0.95416549
LAMHUP	5	0.89748127
LAMHUP	5	0.96833655
LAMHUP	5	0.97109293
LAMHUP	5	1.00227359
LAMHUP	5	1.0143994
LAMHUP	5	0.94770744
LAMHUP	10	0.35267857
LAMHUP	10	0.53521825
LAMHUP	10	0.59474206
LAMHUP	10	0.56299603
LAMHUP	10	0.98073622
LAMHUP	10	0.92759476
LAMHUP	10	1.00553557
LAMHUP	10	0.97719346
LAMHUP	10	0.79786707
LAMHUP	10	0.69306542
LAMHUP	10	0.65495573
LAMHUP	10	0.70259284
LAMHUP	30	0.56299603
LAMHUP	30	0.33680556

Cell	Dose [µM]	Response
LAMHUP	30	0.92013889
LAMHUP	30	0.75744048
LAMHUP	30	0.6990865
LAMHUP	30	0.68137268
LAMHUP	30	0.51663416
LAMHUP	30	0.70085788
LAMHUP	30	0.37432983
LAMHUP	30	0.19244268
LAMHUP	30	0.8931413
LAMHUP	30	0.48606051
LAMHUP	45	0.28125
LAMHUP	45	0.15029762
LAMHUP	45	0.05902778
LAMHUP	45	0.15029762
LAMHUP	45	0.69377235
LAMHUP	45	0.68491544
LAMHUP	45	0.59988911
LAMHUP	45	0.66188748
LAMHUP	45	0.49039116
LAMHUP	45	0.3379524
LAMHUP	45	0.3674008
LAMHUP	45	0.43495888
LAMHUP	50	0.08680556
LAMHUP	50	0.06696429

Cell	Dose [µM]	Response
LAMHUP	50	0.16617063
LAMHUP	50	0.09474206
NHLF	0	1.033543
NHLF	0	0.97284614
NHLF	0	0.97284614
NHLF	0	0.96965157
NHLF	0	1.04951586
NHLF	0	0.92492756
NHLF	0	0.93770585
NHLF	0	1.13896387
NHLF	0	0.75667968
NHLF	0	0.89751625
NHLF	0	1.21439853
NHLF	0	0.98302416
NHLF	0	1.1087711
NHLF	0	0.98805404
NHLF	0	1.27475706
NHLF	0	0.77679919
NHLF	0	0.89507687
NHLF	0	0.8497047
NHLF	0	1.23788878
NHLF	0	1.03875539
NHLF	0	0.98834187
NHLF	0	1.02615201

Cell	Dose [µM]	Response
NHLF	0	1.01354863
NHLF	0	0.95053174
NHLF	0	0.95993037
NHLF	0	0.98276207
NHLF	0	0.98093553
NHLF	0	0.94623135
NHLF	0	1.06312964
NHLF	0	1.00011416
NHLF	0	1.05491023
NHLF	0	1.01198664
NHLF	0	0.97047469
NHLF	0	1.03834897
NHLF	0	0.99898189
NHLF	0	0.96640223
NHLF	0	1.00169686
NHLF	0	0.96097229
NHLF	0	0.99490943
NHLF	0	1.06821366
NHLF	0	0.92684442
NHLF	0	1.02770291
NHLF	0	1.03035708
NHLF	0	1.10201969
NHLF	0	0.99452577
NHLF	0	1.02372165

Cell	Dose [µM]	Response
NHLF	0	1.00646954
NHLF	0	0.88835894
NHLF	1	0.88247349
NHLF	1	0.87995281
NHLF	1	0.91524227
NHLF	1	0.94044903
NHLF	1	1.04943062
NHLF	1	0.9818488
NHLF	1	1.06678271
NHLF	1	0.96814978
NHLF	1	1.00169686
NHLF	1	0.94468246
NHLF	1	1.01527171
NHLF	1	0.90667286
NHLF	1	0.97594658
NHLF	1	1.0011612
NHLF	1	0.92286316
NHLF	1	0.95471321
NHLF	5	0.74283697
NHLF	5	0.5703301
NHLF	5	0.57991381
NHLF	5	0.79714469
NHLF	5	0.3945285
NHLF	5	0.16818401

Cell	Dose [µM]	Response
NHLF	5	0.5504547
NHLF	5	0.63093274
NHLF	5	0.82701862
NHLF	5	0.82449794
NHLF	5	0.85474605
NHLF	5	0.95305241
NHLF	5	0.9562773
NHLF	5	1.01563971
NHLF	5	0.95810384
NHLF	5	0.89417509
NHLF	5	0.84965846
NHLF	5	0.86187583
NHLF	5	0.9121028
NHLF	5	0.9596148
NHLF	5	0.95736738
NHLF	5	0.85783597
NHLF	5	0.93215276
NHLF	5	1.0104508
NHLF	10	0.57991381
NHLF	10	0.7620044
NHLF	10	0.6374161
NHLF	10	0.78117183
NHLF	10	0.72650041
NHLF	10	0.37943887

Cell	Dose [µM]	Response
NHLF	10	0.3945285
NHLF	10	0.39955838
NHLF	10	0.81441524
NHLF	10	0.79677051
NHLF	10	0.82701862
NHLF	10	0.80181186
NHLF	10	0.76723085
NHLF	10	0.71700112
NHLF	10	0.79097582
NHLF	10	0.80102176
NHLF	10	0.87273571
NHLF	10	0.81436383
NHLF	10	0.7695668
NHLF	10	0.81572131
NHLF	10	0.72247326
NHLF	10	0.80607964
NHLF	10	0.85252763
NHLF	10	0.7556504
NHLF	15	0.47449294
NHLF	15	0.35629379
NHLF	15	0.40101779
NHLF	15	0.55435724
NHLF	15	0.27381144
NHLF	15	0.32914009

Cell	Dose [µM]	Response
NHLF	15	0.49512605
NHLF	15	0.26375169
NHLF	30	0.54157895
NHLF	30	0.48088209
NHLF	30	0.68214011
NHLF	30	0.61185953
NHLF	30	0.69129127
NHLF	30	0.78182906
NHLF	30	0.2386023
NHLF	30	0.56554433
NHLF	30	0.80433254
NHLF	30	0.77156375
NHLF	30	0.79424983
NHLF	30	0.86734943
NHLF	30	0.79188909
NHLF	30	0.57087826
NHLF	30	0.74439916
NHLF	30	0.61928146
NHLF	30	0.86730577
NHLF	30	0.77499674
NHLF	30	0.76549434
NHLF	30	0.79943148
NHLF	30	0.15315362
NHLF	30	0.71451075

Cell	Dose [µM]	Response
NHLF	30	0.73441703
NHLF	30	0.90826522
NHLF	45	0.30837521
NHLF	45	0.28281863
NHLF	45	0.41060151
NHLF	45	0.46171465
NHLF	45	0.08770597
NHLF	45	0.40961813
NHLF	45	-0.1839074
NHLF	45	0.10782548
NHLF	45	0.74887767
NHLF	45	0.68838145
NHLF	45	0.71862956
NHLF	45	0.75139834
NHLF	45	0.49142396
NHLF	45	0.47498514
NHLF	45	0.50877605
NHLF	45	0.52338833
NHLF	45	0.46141754
NHLF	45	0.44377023
NHLF	45	0.57137388
NHLF	45	0.55236908
NHLF	45	0.41193528
NHLF	45	0.51412085

Cell	Dose [µM]	Response
NHLF	45	0.54066256
NHLF	45	0.50881251
NHLF	50	0.45532551
NHLF	50	0.49685494
NHLF	50	0.53838438
NHLF	50	0.46171465
NHLF	50	0
NHLF	50	0
NHLF	50	0.13297487
NHLF	50	0
NHLF	100	0.08156061
NHLF	100	0.17100862
NHLF	100	0.25726206
NHLF	100	0.27323492
NHLF	100	0
NHLF	100	0
NHLF	100	0
NHLF	100	0
TTJ_TSC2-	0	0.75358094
TTJ_TSC2-	0	0.8854039
TTJ_TSC2-	0	1.14305787
TTJ_TSC2-	0	0.95730734
TTJ_TSC2-	0	1.01123491
TTJ_TSC2-	0	1.11010213

Cell	Dose [µM]	Response
TTJ_TSC2-	0	1.06516249
TTJ_TSC2-	0	1.07415042
TTJ_TSC2-	0	1.05693973
TTJ_TSC2-	0	1.0162685
TTJ_TSC2-	0	0.8454493
TTJ_TSC2-	0	1.31723566
TTJ_TSC2-	0	1.08947672
TTJ_TSC2-	0	0.91052328
TTJ_TSC2-	0	0.95119451
TTJ_TSC2-	0	0.8129123
TTJ_TSC2-	0	0.933686
TTJ_TSC2-	0	0.81328613
TTJ_TSC2-	0	1.09171084
TTJ_TSC2-	0	1.20082323
TTJ_TSC2-	0	1.02774841
TTJ_TSC2-	0	1.02398591
TTJ_TSC2-	0	0.97131097
TTJ_TSC2-	0	0.9374485
TTJ_TSC2-	5	0.75657692
TTJ_TSC2-	5	0.87042402
TTJ_TSC2-	5	0.8854039
TTJ_TSC2-	5	0.89439183
TTJ_TSC2-	5	0.95249849
TTJ_TSC2-	5	0.65526129

Cell	Dose [µM]	Response
TTJ_TSC2-	5	0.92239852
TTJ_TSC2-	5	0.77566116
TTJ_TSC2-	10	0.84046426
TTJ_TSC2-	10	0.99325905
TTJ_TSC2-	10	0.76556485
TTJ_TSC2-	10	0.69965337
TTJ_TSC2-	10	0.42951152
TTJ_TSC2-	10	1.61846031
TTJ_TSC2-	10	0.440799
TTJ_TSC2-	10	0.8358611
TTJ_TSC2-	15	0.78653668
TTJ_TSC2-	15	0.68167751
TTJ_TSC2-	15	1.00823894
TTJ_TSC2-	15	0.60378212
TTJ_TSC2-	15	0.89229855
TTJ_TSC2-	15	0.66278628
TTJ_TSC2-	15	0.31287413
TTJ_TSC2-	15	0.50099894
TTJ_TSC2-	30	0.18434542
TTJ_TSC2-	30	0.04653051
TTJ_TSC2-	30	0.31916436
TTJ_TSC2-	30	0.11543797
TTJ_TSC2-	30	-0.4860021
TTJ_TSC2-	30	-0.5200327

Cell	Dose [µM]	Response
TTJ_TSC2-	30	-0.4973456
TTJ_TSC2-	30	-0.3725668
TTJ_TSC2-	45	0.12142992
TTJ_TSC2-	45	0.09746211
TTJ_TSC2-	45	0.08248223
TTJ_TSC2-	45	0.0734943
TTJ_TSC2-	45	-0.5313762
TTJ_TSC2-	45	-0.5540633
TTJ_TSC2-	45	-0.5313762
TTJ_TSC2-	45	-0.5313762
TTJ_TSC2-	50	0.0854782
TTJ_TSC2-	50	0.07649027
TTJ_TSC2-	50	0.11543797
TTJ_TSC2-	50	-0.0073971
TTJ_TSC2-	50	-0.1631974
TTJ_TSC2-	50	-0.0086467
TTJ_TSC2-	50	-0.3014796
TTJ_TSC2-	50	-0.1631974
TTJ_TSC2-	50	-0.3725668
TTJ_TSC2-	50	-0.440628
TTJ_TSC2-	50	-0.4292845
TTJ_TSC2-	50	-0.2137574
TTJ_TSC2-	100	-0.4397618
TTJ_TSC2-	100	-0.3665536

Cell	Dose [µM]	Response
TTJ_TSC2-	100	-0.1713317
TTJ_TSC2-	100	-0.3909563
TTJ_TSC2-	100	-0.0433488
TTJ_TSC2-	100	-0.0942804
TTJ_TSC2-	100	0.01956672
TTJ_TSC2-	100	0.02555867
TTJ_TSC2-	100	-0.4179409
TTJ_TSC2-	100	-0.4746586
TTJ_TSC2-	100	-0.247788
TTJ_TSC2-	100	-0.2137574
TTJ_TSC2+	0	0.80686267
TTJ_TSC2+	0	0.99782992
TTJ_TSC2+	0	1.14105535
TTJ_TSC2+	0	0.98914959
TTJ_TSC2+	0	0.93272745
TTJ_TSC2+	0	1.10633404
TTJ_TSC2+	0	1.00651025
TTJ_TSC2+	0	1.01953074
TTJ_TSC2+	0	0.98346583
TTJ_TSC2+	0	1.03434019
TTJ_TSC2+	0	1.0207737
TTJ_TSC2+	0	0.98346583
TTJ_TSC2+	0	1.01399045
TTJ_TSC2+	0	0.95972446

Cell	Dose [µM]	Response
TTJ_TSC2+	0	1.01059883
TTJ_TSC2+	0	0.9936407
TTJ_TSC2+	0	0.84516129
TTJ_TSC2+	0	0.77634409
TTJ_TSC2+	0	1.22365591
TTJ_TSC2+	0	0.9827957
TTJ_TSC2+	0	0.9655914
TTJ_TSC2+	0	0.9311828
TTJ_TSC2+	0	1.17204301
TTJ_TSC2+	0	1.10322581
TTJ_TSC2+	5	1.18696328
TTJ_TSC2+	5	0.87154224
TTJ_TSC2+	5	0.81388463
TTJ_TSC2+	5	0.92580822
TTJ_TSC2+	5	0.38064516
TTJ_TSC2+	5	0.51827957
TTJ_TSC2+	5	0.75913978
TTJ_TSC2+	5	0.48387097
TTJ_TSC2+	10	0.79014326
TTJ_TSC2+	10	0.72909403
TTJ_TSC2+	10	0.68500292
TTJ_TSC2+	10	0.72231078
TTJ_TSC2+	10	0.46666667
TTJ_TSC2+	10	0.43225806

Cell	Dose [µM]	Response
TTJ_TSC2+	10	0.36344086
TTJ_TSC2+	10	0.51827957
TTJ_TSC2+	15	0.87832549
TTJ_TSC2+	15	0.77318514
TTJ_TSC2+	15	0.88850036
TTJ_TSC2+	15	0.79353489
TTJ_TSC2+	15	-0.0666667
TTJ_TSC2+	15	0.12258065
TTJ_TSC2+	15	0.10537634
TTJ_TSC2+	15	0.29462366
TTJ_TSC2+	30	0.17625931
TTJ_TSC2+	30	0.90545848
TTJ_TSC2+	30	0.0202446
TTJ_TSC2+	30	0.03041948
TTJ_TSC2+	30	-0.7892473
TTJ_TSC2+	30	-0.8580645
TTJ_TSC2+	30	-0.7376344
TTJ_TSC2+	30	-0.6172043
TTJ_TSC2+	45	0.10503521
TTJ_TSC2+	45	0.08468546
TTJ_TSC2+	45	0.05076922
TTJ_TSC2+	45	0.07790221
TTJ_TSC2+	45	-1.0301075
TTJ_TSC2+	45	-0.8924731

Cell	Dose [µM]	Response
TTJ_TSC2+	45	-0.7892473
TTJ_TSC2+	45	-0.9096774
TTJ_TSC2+	50	-0.05249
TTJ_TSC2+	50	-0.0438096
TTJ_TSC2+	50	0.02129285
TTJ_TSC2+	50	-0.0177686
TTJ_TSC2+	50	0.07451059
TTJ_TSC2+	50	0.05416084
TTJ_TSC2+	50	0.00667811
TTJ_TSC2+	50	0.06094409
TTJ_TSC2+	50	-0.772043
TTJ_TSC2+	50	-0.6516129
TTJ_TSC2+	50	-0.7892473
TTJ_TSC2+	50	-0.427957
TTJ_TSC2+	100	-0.0611703
TTJ_TSC2+	100	-0.0221088
TTJ_TSC2+	100	-0.0741908
TTJ_TSC2+	100	-0.0177686
TTJ_TSC2+	100	0.09486033
TTJ_TSC2+	100	0.12877657
TTJ_TSC2+	100	0.0473776
TTJ_TSC2+	100	0.0338111
TTJ_TSC2+	100	-0.9784946
TTJ_TSC2+	100	-0.9268817

Cell	Dose [µM]	Response
TTJ_TSC2+	100	-0.9096774
TTJ_TSC2+	100	-0.8236559

Supplementary Table S2: Viability Raw Data

Treatment	pcell_621102	pcell_621103	pcell_LAMD100	pcell_NHLF	pcell_TTJTSC2-	pcell_TTJTSC2+
0.1% DMSO	102.3125922	100.9619405	98.36207473	99.7848755	95.81966414	97.1987935
0.1% DMSO	98.25686047	98.64750864	96.74427745	101.968117	104.1851161	102.802951
0.1% DMSO	99.43054734	100.4011482	104.8939313	98.2475735	n.a.	n.a.
10 nM Rapamycin	101.5243596	100.7095056	103.0867699	89.9256162	102.8720982	105.037406
10 nM Rapamycin	97.08964168	98.68488928	104.5097044	103.577604	106.7550156	103.848462
10 nM Rapamycin	96.26684811	97.00541788	107.7429022	103.968597	n.a.	n.a.
10 uM NTZ	98.85989452	98.21271278	102.4506188	90.1844364	92.0301859	101.948343
10 uM NTZ	97.38437371	99.74559075	93.49449946	95.0157455	104.5462912	105.35095
10 uM NTZ	94.66854795	100.025315	97.34156821	95.0157455	n.a.	n.a.
27 uM NTZ	101.2584625	98.58183995	104.2233764	89.2515809	99.39260077	108.967752
27 uM NTZ	97.58043083	99.54729682	98.50908327	91.443725	105.9286221	107.354909
27 uM NTZ	94.66854795	95.31289777	99.19865939	73.7588536	n.a.	n.a.
45 uM NTZ	95.33673363	86.25017596	86.40767406	78.727332	91.22992341	108.172851
45 uM NTZ	102.506366	78.94049708	94.93430546	62.7405557	85.89484017	93.0711191
45 uM NTZ	96.6782449	94.04849375	85.77992601	78.4256947	n.a.	n.a.

Values shown are percentages, calculated according to average of 0.1% DMSO treatment group.

Supplementary Table S3: Subcutaneous Injection Tumor Volume [mm^3]

Group	Day 1	Day 3	Day 5	Day 8	Day 10	Day 12	Day 15	Day 17	Day 19	Day 22	Day 24
Untreated	81.6	144.9	151.6	312.7	465.5	580.0	853.0	896.6	868.6	1026.7	1402.6
Untreated	26.0	91.0	94.2	239.1	370.4	355.2	525.0	540.0	571.4	641.6	560.0
Untreated	66.3	80.0	85.7	127.1	232.7	241.1	483.8	565.0	830.0	760.3	613.8
Untreated	26.0	81.5	94.1	178.6	234.4	284.6	517.5	1318.7	1282.8	1548.0	1964.9
Untreated	13.5	80.1	84.0	334.1	460.8	463.4	645.0	761.4	801.4	1023.4	1098.1
Untreated	87.7	143.4	129.6	261.6	366.1	375.6	500.0	787.2	624.2	953.1	792.6
Untreated	55.0	151.6	144.2	289.7	415.4	575.0	661.5	802.8	838.2	863.8	922.8
Untreated	37.9	52.9	66.3	96.9	163.8	183.3	384.8	387.2	540.0	598.2	863.8
Untreated	78.7	132.1	152.8	327.7	442.8	530.2	872.9	836.4	882.9	742.6	766.2
Rapamycin (4mg/kg)	43.6	147.6	123.9	119.4	88.1	99.1	45.0	39.8	16.4	10.8	6.0
Rapamycin (4mg/kg)	27.4	51.8	50.8	108.2	118.4	134.9	59.0	56.4	29.8	8.6	4.8
Rapamycin (4mg/kg)	50.6	81.3	100.4	112.1	89.2	77.2	38.3	22.5	18.4	14.7	13.5
Rapamycin (4mg/kg)	60.8	111.0	106.8	56.0	39.3	49.4	27.6	8.2	6.0	1.0	0.8
Rapamycin (4mg/kg)	89.2	90.0	88.8	46.9	43.3	35.0	16.0	14.2	15.8	7.5	6.0
Rapamycin (4mg/kg)	92.5	104.4	90.8	45.3	16.7	22.9	8.2	6.1	4.6	4.0	0.5
Rapamycin (4mg/kg)	34.2	81.3	86.5	220.3	176.4	203.4	119.1	77.2	39.5	19.6	16.8
Rapamycin (4mg/kg)	32.7	102.5	145.8	76.7	81.0	68.8	36.1	22.1	7.4	7.5	5.1
Rapamycin (4mg/kg)	25.3	126.0	143.7	107.9	95.8	81.6	32.8	24.6	16.4	8.4	5.5
Rapamycin (4mg/kg)	33.2	73.0	80.2	109.3	90.4	85.2	46.5	46.6	30.0	20.1	13.3
NTZ (50mg/kg)	60.8	68.8	98.3	246.4	457.7	508.1	600.0	678.0	0.0	0.0	0.0
NTZ (50mg/kg)	32.0	73.0	87.7	368.6	488.4	662.9	730.7	697.1	865.5	1482.8	1538.6
NTZ (50mg/kg)	34.4	41.5	42.8	101.3	196.2	193.2	423.4	296.5	352.3	593.0	607.0
NTZ (50mg/kg)	63.0	58.5	56.1	126.7	252.5	294.4	368.6	354.8	594.1	835.4	869.3
NTZ (50mg/kg)	13.5	35.3	34.5	61.7	110.9	169.0	253.1	307.2	457.2	1046.2	1053.4

Group	Day 1	Day 3	Day 5	Day 8	Day 10	Day 12	Day 15	Day 17	Day 19	Day 22	Day 24
NTZ (50mg/kg)	18.9	27.4	28.9	49.6	100.9	81.1	114.5	137.7	204.8	291.2	321.0
NTZ (50mg/kg)	28.1	51.6	51.6	87.1	105.9	138.6	171.2	251.4	347.9	600.0	650.0
NTZ (50mg/kg)	37.0	62.5	81.6	181.5	307.1	299.9	530.5	550.0	733.8	1167.1	1461.3
NTZ (50mg/kg)	13.5	55.7	71.3	231.2	321.7	308.4	416.3	595.0	647.1	1022.4	1026.7
NTZ (50mg/kg)	64.5	87.8	78.4	105.0	132.3	255.5	491.9	423.4	440.1	834.2	1056.8
NTZ (500mg/kg) proph.	62.5	73.0	89.4	147.9	262.4	333.0	459.6	482.0	523.4	707.9	848.7
NTZ (500mg/kg) proph.	32.0	77.5	86.3	210.0	301.2	280.1	515.0	505.0	470.5	274.4	360.1
NTZ (500mg/kg) proph.	56.7	70.2	83.2	205.4	210.9	269.0	389.2	520.0	539.1	546.4	465.7
NTZ (500mg/kg) proph.	37.0	44.8	86.2	175.7	190.4	231.8	319.4	423.8	454.1	376.5	490.1
NTZ (500mg/kg) proph.	36.0	72.5	78.8	98.3	131.2	186.3	203.4	251.3	238.2	262.1	340.7
NTZ (500mg/kg) proph.	4.0	6.1	19.6	102.5	117.5	147.4	201.1	202.0	179.6	180.3	201.6
NTZ (500mg/kg) proph.	41.6	15.8	37.5	90.0	97.5	134.6	162.5	198.0	305.3	570.0	600.0
NTZ (500mg/kg) proph.	19.8	32.0	64.5	215.3	396.8	480.1	691.5	843.4	854.0	1216.8	1391.5
NTZ (500mg/kg) proph.	28.9	35.2	52.7	138.1	192.2	217.6	272.2	310.0	327.7	423.2	388.3
NTZ (500mg/kg) proph.	83.2	121.1	129.0	299.9	500.0	499.9	480.0	206.5	232.7	258.8	268.6

Supplementary Table S4: Tail Vein Injection Lesions

Group	lesion_pixels	lung_pixels	percent
Rapamycin (0.5 mg/kg) i.p.	408	397307	0.10269137
Rapamycin (0.5 mg/kg) i.p.	97	561951	0.01726129
Rapamycin (0.5 mg/kg) i.p.	120	563574	0.02129268
Rapamycin (0.5 mg/kg) i.p.	305	262814	0.11605166
Rapamycin (0.5 mg/kg) i.p.	3969	365701	1.08531286
Rapamycin (0.5 mg/kg) i.p.	367	282159	0.13006851
Rapamycin (0.5 mg/kg) i.p.	28	413799	0.00676657
Rapamycin (0.5 mg/kg) i.p.	77	385508	0.01997365
Rapamycin (0.5 mg/kg) i.p.	18747	482076	3.88880591
Rapamycin (0.5 mg/kg) i.p.	8161	491182	1.66150225
NTZ (500 mg/kg) oral + Rapa (0.5 mg/kg) i.p.	3054	477391	0.63972718
NTZ (500 mg/kg) oral + Rapa (0.5 mg/kg) i.p.	4513	398231	1.13326185
NTZ (500 mg/kg) oral + Rapa (0.5 mg/kg) i.p.	1757	338123	0.51963339
NTZ (500 mg/kg) oral + Rapa (0.5 mg/kg) i.p.	46955	551173	8.5191038
NTZ (500 mg/kg) oral + Rapa (0.5 mg/kg) i.p.	441	341030	0.12931414
NTZ (500 mg/kg) oral + Rapa (0.5 mg/kg) i.p.	160	376053	0.04254719
NTZ (500 mg/kg) oral + Rapa (0.5 mg/kg) i.p.	661	410501	0.16102275
NTZ (500 mg/kg) oral + Rapa (0.5 mg/kg) i.p.	25794	463860	5.56072953
NTZ (500 mg/kg) oral + Rapa (0.5 mg/kg) i.p.	62	458247	0.01352982
NTZ (500 mg/kg) oral + Rapa (0.5 mg/kg) i.p.	172	451429	0.03810123
NTZ (500 mg/kg) oral	23300	482408	4.82993649
NTZ (500 mg/kg) oral	133935	487902	27.4512095
NTZ (500 mg/kg) oral	32402	395058	8.20183365
NTZ (500 mg/kg) oral	51433	442464	11.6242225

Group	lesion_pixels	lung_pixels	percent
NTZ (500 mg/kg) oral	11535	360477	3.19992676
NTZ (500 mg/kg) oral	169175	420384	40.2429683
NTZ (500 mg/kg) oral	98759	427074	23.1245639
NTZ (500 mg/kg) oral	0	515083	0
NTZ (500 mg/kg) oral	252080	477650	52.7750445
NTZ (500 mg/kg) oral	100073	423505	23.6297092
Vehicle	4861252	10520489	46.21
Vehicle	5158441	7952138	64.87
Vehicle	3761363	8364817	44.97
Vehicle	3668435	9268590	39.58
Vehicle	4907124	11936471	41.11
NTZ (100 mg/kg) i.p.	1549606	8699927	17.81
NTZ (100 mg/kg) i.p.	0	7476308	0.00
NTZ (100 mg/kg) i.p.	0	10410995	0.00
NTZ (100 mg/kg) i.p.	1415494	7715611	18.35
NTZ (100 mg/kg) i.p.	6041586	12733044	47.45
Rapamycin (0.1 mg/kg) i.p.	369896	7701330	4.80
Rapamycin (0.1 mg/kg) i.p.	4616766	11429960	40.39
Rapamycin (0.1 mg/kg) i.p.	16028	8635938	0.19
Rapamycin (0.1 mg/kg) i.p.	5807959	11451805	50.72
Rapamycin (0.1 mg/kg) i.p.	4612889	9063899	50.89
NTZ (100 mg/kg) i.p. + Rapa (0.1 mg/kg) i.p.	6142741	15863948	38.72
NTZ (100 mg/kg) i.p. + Rapa (0.1 mg/kg) i.p.	0	7034965	0.00
NTZ (100 mg/kg) i.p. + Rapa (0.1 mg/kg) i.p.	0	4610982	0.00
NTZ (100 mg/kg) i.p. + Rapa (0.1 mg/kg) i.p.	2851876	12312971	23.16
