# Supplementary figures and images for: Repurposing Nitazoxanide for Potential Treatment of Rare Disease Lymphangioleiomyomatosis
Source: Biomolecules. 2024 Sep 30;14(10):1236. doi: 10.3390/biom14101236 (PMC11506457; doi:10.3390/biom14101236)

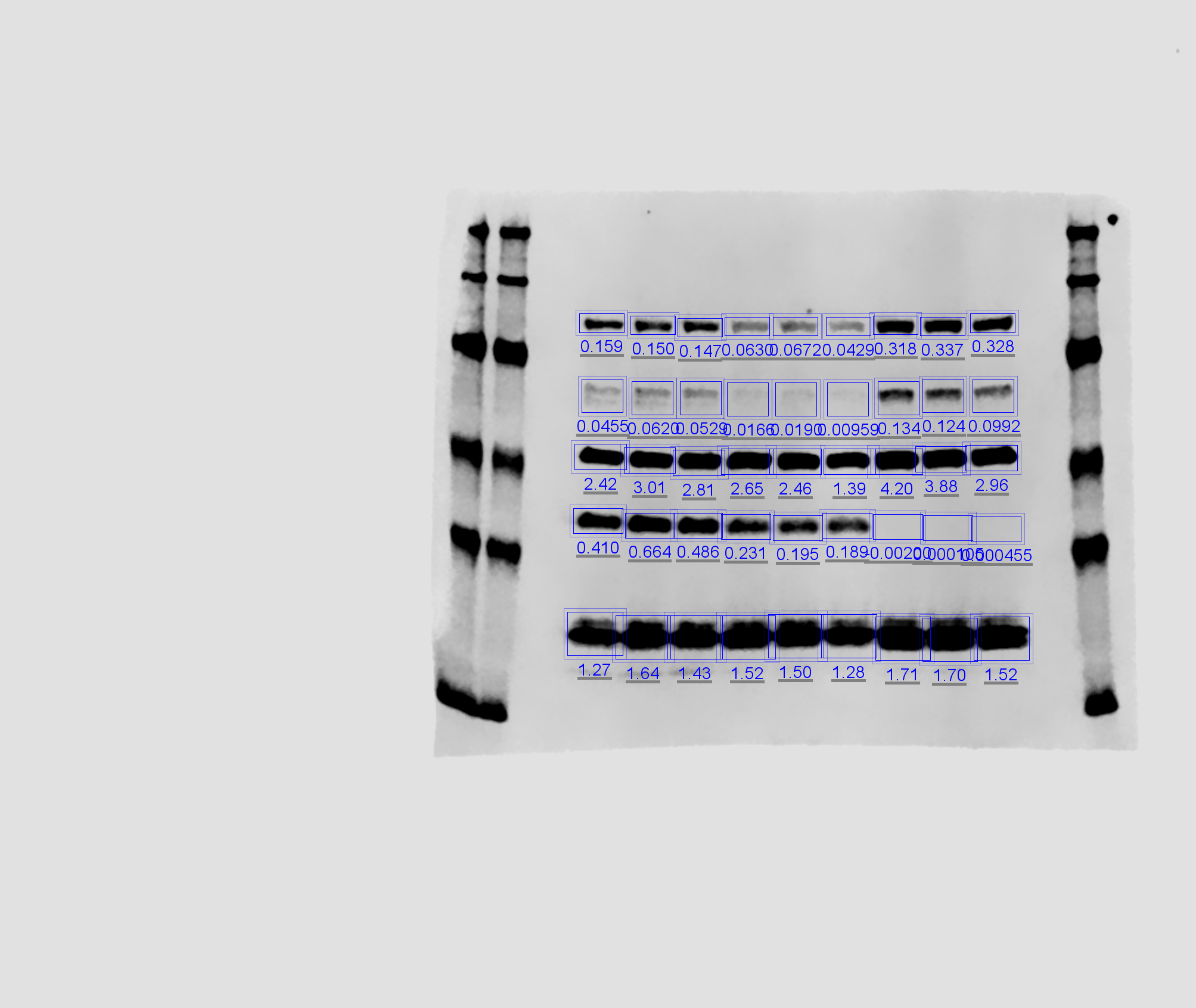

Supplement: Supplementary file 1 [file biomolecules-14-01236-s001.zip › Western_Blot_Quantified.tif]

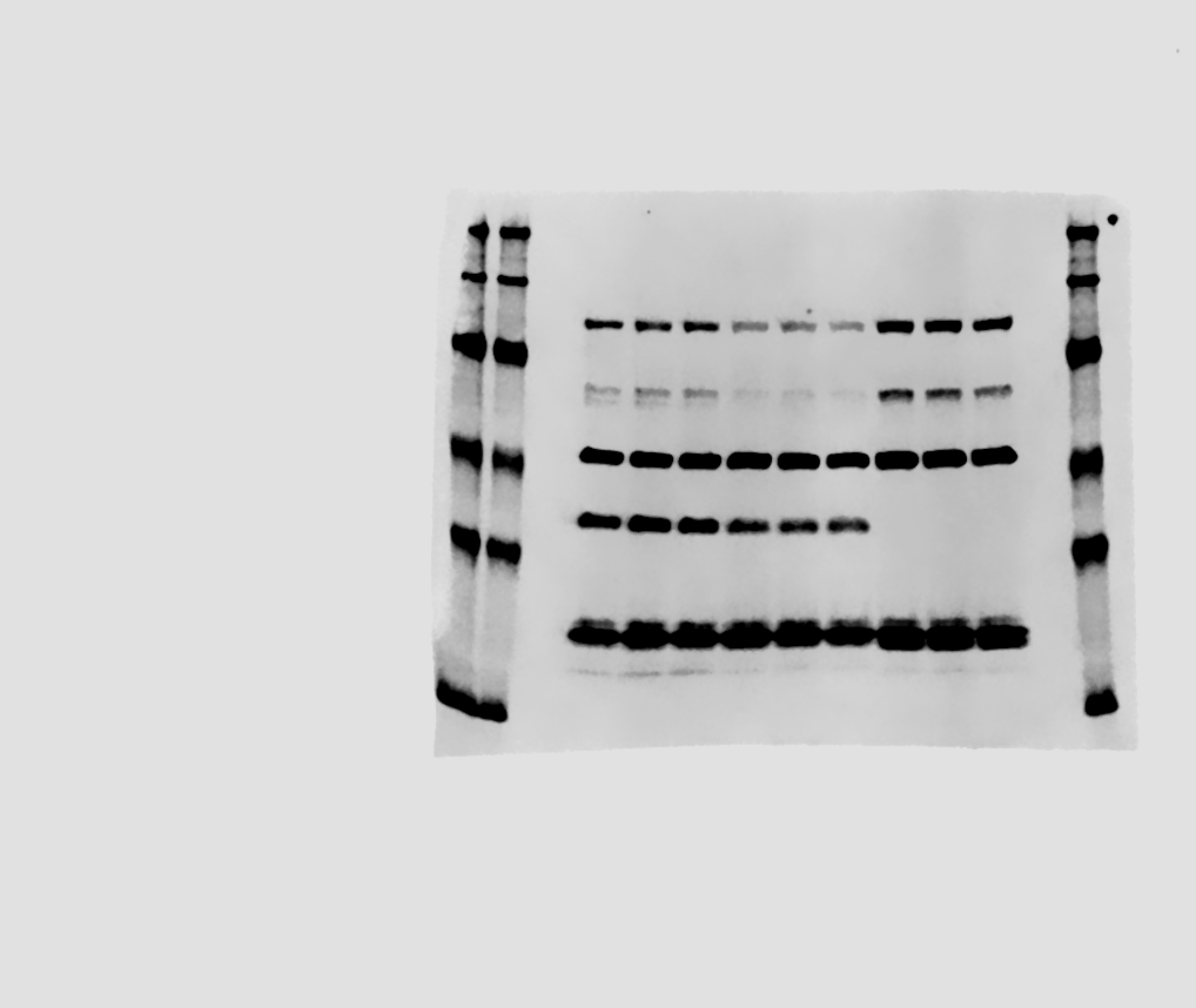

Supplement: Supplementary file 1 [file biomolecules-14-01236-s001.zip › Western_Blot_Raw.tif]
